# Microglial deactivation by adeno‐associated virus expressing small‐hairpin GCH1 has protective effects against neuropathic pain development in a spinothalamic tract‐lesion model

**DOI:** 10.1111/cns.13751

**Published:** 2021-11-29

**Authors:** Hyun Ho Jung, Chin Su Koh, Minkyung Park, Ji Hyun Kim, Ha‐Na Woo, Heuiran Lee, Jin Woo Chang

**Affiliations:** ^1^ Department of Neurosurgery Yonsei University College of Medicine Seoul Korea; ^2^ Brain Korea 21 PLUS Project for Medical Science and Brain Research Institute Yonsei University College of Medicine Seoul Korea; ^3^ Department of Microbiology University of Ulsan College of Medicine Seoul Korea; ^4^ Bio‐Medical Institute of Technology University of Ulsan College of Medicine Seoul Korea; ^5^ Department of Biochemistry & Molecular Biology University of Ulsan College of Medicine Seoul Korea; ^6^ Department of Microbiology, Asan Medical Center University of Ulsan College of Medicine Seoul Korea

**Keywords:** microglial deactivation, neuropathic pain, rAAV‐shGCH1, spinal cord injury

## Abstract

**Aims:**

Neuropathic pain after spinal cord injury is one of the most difficult clinical problems after the loss of mobility, and pharmacological or neuromodulation therapy showed limited efficacy. In this study, we examine the possibility of pain modulation by a recombinant adeno‐associated virus (rAAV) encoding small‐hairpin RNA against GCH1 (rAAV‐shGCH1) in a spinal cord injury model in which neuropathic pain was induced by a spinothalamic tract (STT) lesion.

**Methods:**

Micro‐electric lesioning was used to damage the left STT in rats (*n* = 32), and either rAAV‐shGCH1 (*n* = 19) or rAAV control (*n* = 6) was injected into the dorsal horn of the rats at the same time. On postoperative days 3, 7, and 14, we evaluated neuropathic pain using a behavioral test and microglial activation by immunohistochemical staining.

**Results:**

A pain modulation effect of shGCH1 was observed from postoperative days 3 to 14. The mechanical withdrawal threshold was 13.0 ± 0.95 in the shGCH1 group, 4.3 ± 1.37 in the control group, and 3.49 ± 0.85 in sham on postoperative day 3 (*p* < 0.0001) and continued to postoperative day 14 (shGCH1 vs. control: 11.4 ± 1.1 vs. 2.05 ± 0.60, *p* < 0.001 and shGCH1 vs. sham: 11.4 ± 1.1 vs. 1.43 ± 0.54, *p* < 0.001). Immunohistochemical staining of the spinal cord dorsal horn showed deactivation of microglia in the shGCH1 group without any change of delayed pattern of astrocyte activation as in STT model.

**Conclusions:**

Neuropathic pain after spinal cord injury can be modulated bilaterally by deactivating microglial activation after a unilateral injection of rAAV‐shGCH1 into the dorsal horn of a STT lesion spinal cord pain model. This new attempt would be another therapeutic approach for NP after SCI, which once happens; there is no clear curative options still now.

## Graphical abstract

Neuropathic pain after spinal cord injury can be modulated bilaterally by deactivating microglial activation after a unilateral injection of rAAV‐shGCH1 into the dorsal horn of a spinothalamic tract‐lesion spinal cord pain model.

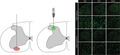

## 1 INTRODUCTION

A recent review by Burke et al. revealed that the prevalence rate of neuropathic pain (NP) after spinal cord injury (SCI) was >50%, representing one of the most difficult clinical problems after the loss of mobility.^1^ Various pharmacological agents, such as opioids, anticonvulsants, antispasmotics, tricyclics, and anesthetics, have been prescribed; however, only a few anticonvulsants, such as gabapentin and pregabalin, have demonstrated strong efficacy.^2^ Combination pharmacological treatments have not shown additional pain reduction either.^3^ In cases of refractory pain, neuromodulation therapies such as spinal cord stimulation, deep brain stimulation, and motor cortex stimulation are considered to have limited efficacy based on pain levels, pain characteristics, and the degree of SCI.^4^, ^5^, ^6^, ^7^ Because of the complexity of individual spinal injuries and the lack of certainty regarding the mechanisms underlying NP after SCI, major obstacles still exist for improving the efficacy of treatment.

Tetrahydrobiopterin (BH4) is an essential cofactor for norepinephrine, dopamine, serotonin, and nitric oxide, all of which have prominent roles in pain signaling.^8^, ^9^, ^10^ Intracellular levels of BH4, which are directly regulated by the rate‐limiting enzyme GTP cyclohydrolase 1 (GCH1), are regulated through de novo synthesis, recycling, and salvage pathways.^11^ In our previous study, a recombinant adeno‐associated virus encoding a small‐hairpin RNA against GCH1 (rAAV‐shGCH1) was injected into the sciatic nerve to investigate this gene therapy against peripheral NP in a spared nerve injury model.^12^ There was an improvement after injection in a pain behavioral test, and microglial activation in the ipsilateral dorsal horn also decreased. The rAAV vector is engineered from a nonpathogenic, non‐enveloped parvovirus that can transduce both dividing and non‐dividing cells.^13^ In vivo delivery of therapeutic rAAV vectors to the retina, liver, and nervous system have resulted in a clinical improvement in patients with congenital blindness, hemophilia B, and spinal muscular atrophy, respectively.^14^ These findings suggest a promising potential for therapeutic rAAV‐based gene therapy applications for NP.

In this study, we examined the possibility of pain modulation by rAAV‐shGCH1 in a SCI model in which NP was induced by creating a lesion in the spinothalamic tract (STT).

## 2 METHODS

### 2.1 Animals

Adult male Sprague Dawley rats (Orientbio) weighing 190–220 g were used in this study. In brief, 2–3 rats were housed in each cage with a 12‐h light/dark cycle and were allowed food and water ad libitum. All experimental procedures were approved by the Institutional Animal Care and Use Committee of Yonsei University Health System and conformed to the guidelines for use of experimental animals published by the International Association for the Study of Pain and the ARRIVE guideline 2.0.^15^ All efforts were made to limit distress and to use a minimal number of animals required to produce scientifically reliable data.

### 2.2 STT lesion to induce NP

**FIGURE 1:**
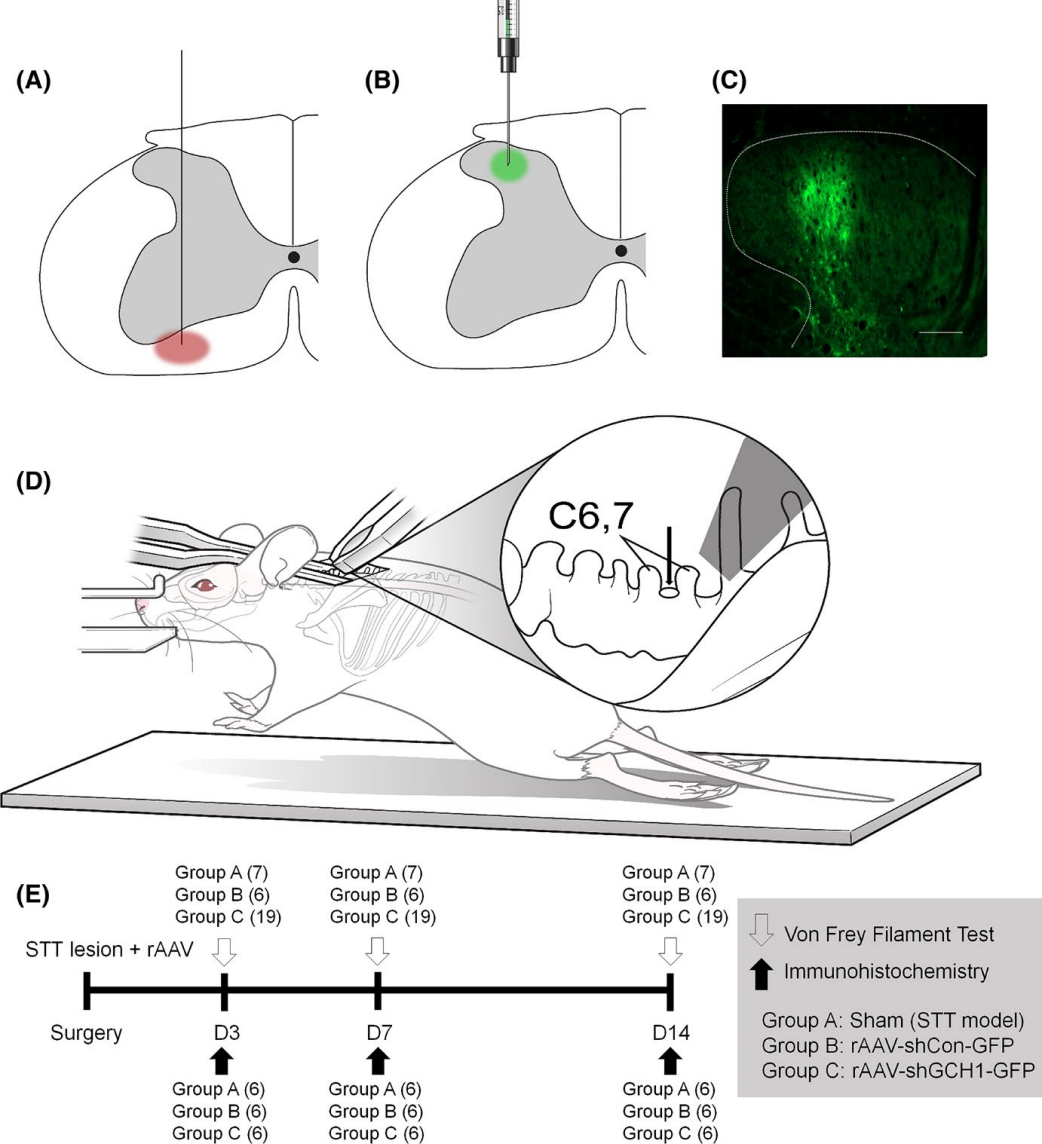
Schematic diagram of spinothalamic tract lesioning and rAAV injection. A, Mirco‐electrode lesioning of the left spinothalamic tract. B, Either rAAV‐shGCH1 or rAAV shCon (3 μl) was injected into the dorsal horn of the rat in the same plane. C, Spinal cord section presents anti‐GFP at the rAAV injection site in the left dorsal horn. D, Surgical diagram for the procedures. E, Overview of the experimental timetable

To induce central NP in these rats, an SCI model using electrical micro‐lesioning was adopted.^16^ The rats (*n* = 68) were anesthetized via an intraperitoneal injection of sodium pentobarbital (40 mg/kg, Hanlim Pharm) following an atropine (0.1 ml, Huons) injection. The tail‐pinch test was used to monitor anesthesia status. After exposure and blunt dissection of the area around C6 and C7, the extraspinal space was exposed by laminectomy according to the methods of Yuta et al.^17^ The left STT was lesioned using a tungsten microelectrode (1 M**Ω**, A‐M systems) that was positioned 0.6–0.8 mm lateral to the midline and 1.8–2.1‐mm deep. A 460‐µA current was applied for 90 s, and a ground electrode was placed in the adjacent muscle (Figure 1A,D).

### 2.3 Preparation of rAAV

**FIGURE 2:**
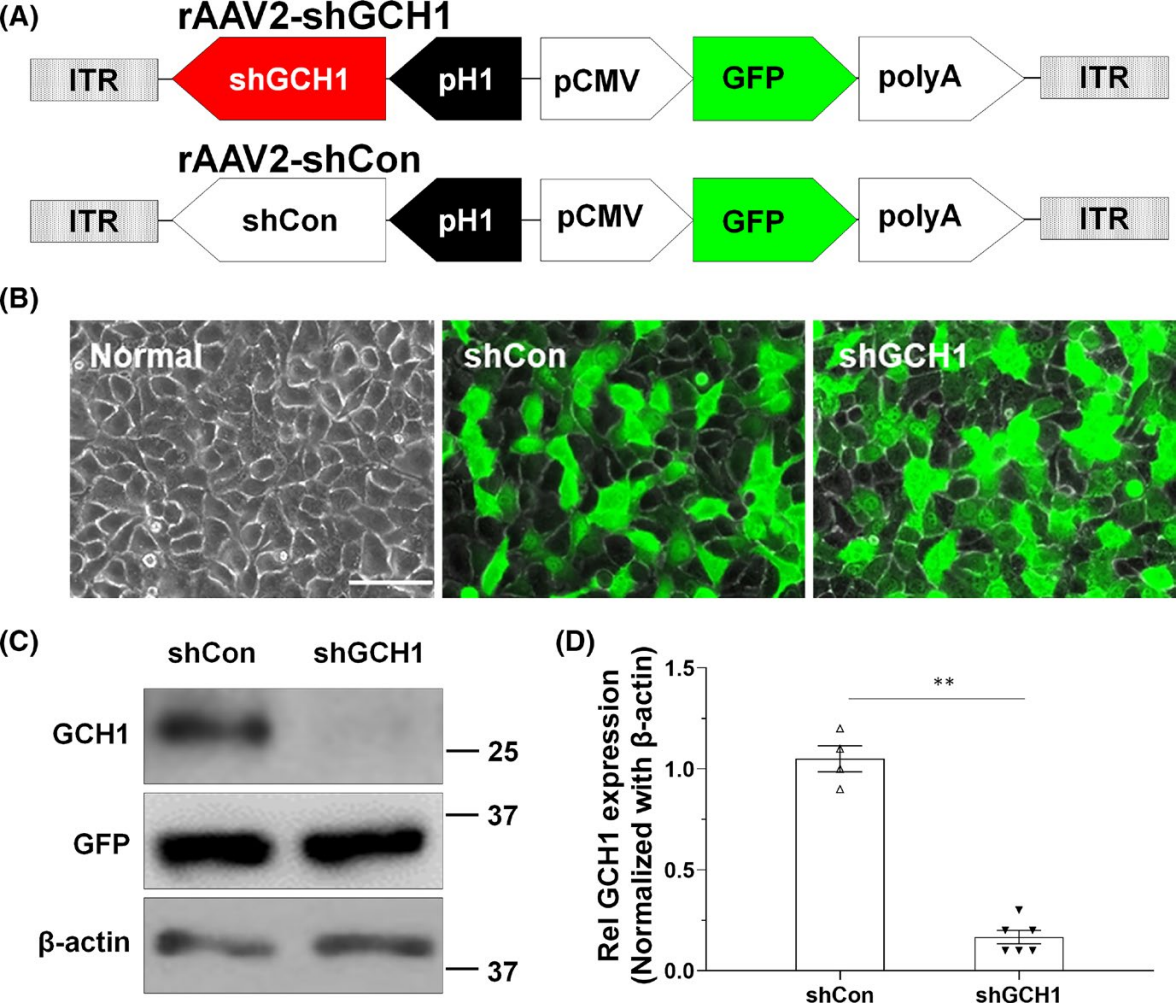
Characteristics of rAAV‐shGCH1. Schematic representation of rAAVs in the present study. A, Short‐hairpin RNA was expressed under the H1 promoter, while the GFP expression cassette was under the control of the CMV promoter with a poly A tail. B, HeLa cells were infected with either rAAV‐shGCH1 or rAAV‐shCon, simultaneously transfecting the cells with pcDNA‐rGCH1 for 48 h. The cells were observed under light/fluorescence microscopy to visualize GFP expression. C, D, Western blotting analysis was then performed. The data showed that rAAV‐shGCH1 effectively downregulated GCH1 expression

The rAAV vector simultaneously expresses short‐hairpin RNA (shRNA) and GFP. The shRNA was expressed under the control of the human H1 polymerase III promoter (pH1), while the opposite orientation GFP expression cassette was under a CMV promoter with a bovine growth hormone polyA signal (Figure 2A). The rAAV vector was produced using a triple co‐transfection method as described previously^1^ and was supplied by CdmoGen Co., Ltd. The number of total virus particles was estimated using real‐time qPCR, as described previously.^18^

### 2.4 Cell culture and in vitro characterization of rAAV

HeLa cells were obtained from the American Type Culture Collection; cultured in Dulbecco's modified Eagle medium (Invitrogen) supplemented with 10% fetal bovine serum (Thermo Fisher Scientific), 2 mM GlutaMAX‐1 (Thermo Fisher Scientific), and penicillin (100 IU/ml)/streptomycin (50 μg/ml); and maintained at 37°C under a humidified 5% carbon dioxide atmosphere. For the characterization for rAAVs, the cells were treated with rAAV at 1000 multiplicity of infection (MOI), together with pcDNA‐rGCH1 complexed with lipofectamine 2000 (Invitrogen). After 48 h, the cell lysates were then prepared. The proteins were resolved on reducing sodium dodecyl sulfate‐polyacrylamide gels and transferred onto polyvinylidene fluoride membranes. Primary antibodies specific to GCH1 (Abnova), GFP (Cell Signaling Tech), and β‐actin (Sigma‐Aldrich) were applied.

### 2.5 AAV injection

After STT lesioning, 3 µl of a solution containing 1.5 × 10^8^ total particles of either rAAV2‐shGCH1‐GFP in 16 rats or rAAV2‐shcon‐GFP in 9 rats was injected to the dorsal horn lesion site (0.6–0.8‐mm lateral to the midline). The syringe needle was kept in place for 3 min after rAAV injection (Figure 1B,C). After completing the combined lesioning and injection procedure, the muscles and skin were approximated and closed in layers by suturing. For sham surgery group (*n* = 7), STT lesioning without any injection was performed. The rats were allowed to recover on a thermoregulated heating pad.

### 2.6 Behavioral test

After recovery from surgery, the mechanical withdrawal threshold for both the left and right hind paws was determined using calibrated von Frey filaments (Stoelting) on postoperative days 3, 7, and 14 (Figure 1E). The rats were placed in hexahedron‐shaped cages (8 × 10 × 12 cm) with a mesh floor. After 30 min of adaptation to minimize anxiety, the filaments were applied to the plantar surface of each paw. The up‐down method was used to gage a response, and pain threshold was then calculated.^19^

### 2.7 Immunohistochemistry

Two weeks after STT lesioning, the animals were transcardially perfused with buffered saline followed by 4% paraformaldehyde. Spinal cord segments from C6 to T1 were carefully dissected, removed, and postfixed for 12 h at 4°C and then placed in a 30% sucrose solution for 72 h at 4°C. Next, 15‐µm‐thick coronal sections were cut using a cryostat microtome and stored in a cryo‐protectant solution (0.1 mol/L PBS, 30% sucrose, 1% polyvinylpyrrolidone, 30% ethylene glycol). Sections were incubated in a blocking solution (5% normal goat serum) with Triton X‐100 (0.3%) for 2 h at room temperature (22 ± 2°C) and then incubated overnight at 4°C with primary antibody polyclonal rabbit anti‐Iba1 (019–19741, 1:300, Wako Chemicals) or mouse anti‐glial fibrillary acidic protein (GFAP) (MAB360, 1:300, Merck) primary antibodies. After incubation, the tissue sections were washed and incubated for 2 h at room temperature in secondary antibody solution (anti‐rabbit Alexa Fluor 488, A11008, 1:500, Invitrogen or anti‐mouse Alexa Fluor 594, A11005, 1:500, Invitrogen). The tissue sections were then washed, slide‐mounted, and subsequently coverslipped with Vectashield hardmount (Vector Laboratories). Overall, 4–5 sections from the C6 to C8 spinal cord segments of each rat were randomly selected and analyzed using an LSM710 Imaging System (Carl Zeiss). Area fractions of microglia were quantified using ImageJ software; http://rsbweb.nih.gov/ij.^20^

### 2.8 Statistical analysis

Data are expressed as means ±standard errors of mean. SPSS 26 (IBM SPSS) and GraphPad Prism 5 (GraphPad Software, Inc.) were used to create graphs and perform all statistical analyses. The Shapiro‐Wilk tests were run for all variables and for the data that failed normality testing, nonparametric test was used. Relative GCH1 expression was compared using the Mann‐Whitney test. Mechanical withdrawal threshold changes and the area fraction of Iba1 and GFAP were analyzed using two‐way analysis of variance (ANOVA) followed by Bonferroni post hoc test, a One‐way ANOVA was used to assess changes in paw withdrawal thresholds both before and after STT lesioning (sham group) and by factoring the two types of rAAV.

## 3 RESULTS

### 3.1 Specific GCH1 downregulation by rAAV‐shGCH1

For indirect monitoring of shRNA expression, a GFP reporter gene was added to the experimental rAAVs (Figure 2A). As expected, a similar degree of GFP expression was observed with equivalent amounts of either virus at the MOI 1000 level (Figure 2B,C, Supplementary Material). In contrast, compared with rAAV‐shCON‐GFP (1.04 ± 0.16, *n* = 4), rAAV‐shGCH1‐GFP dramatically downregulated GCH1 protein level (0.18 ± 0.06, *n* = 6). The data indicate that rAAV‐shGCH1 effectively suppressed GCH1 expression (Figure 2D).

### 3.2 Alleviation of NP behavior after rAAV‐shGCH1‐GFP injection in the STT lesion model

**FIGURE 3:**
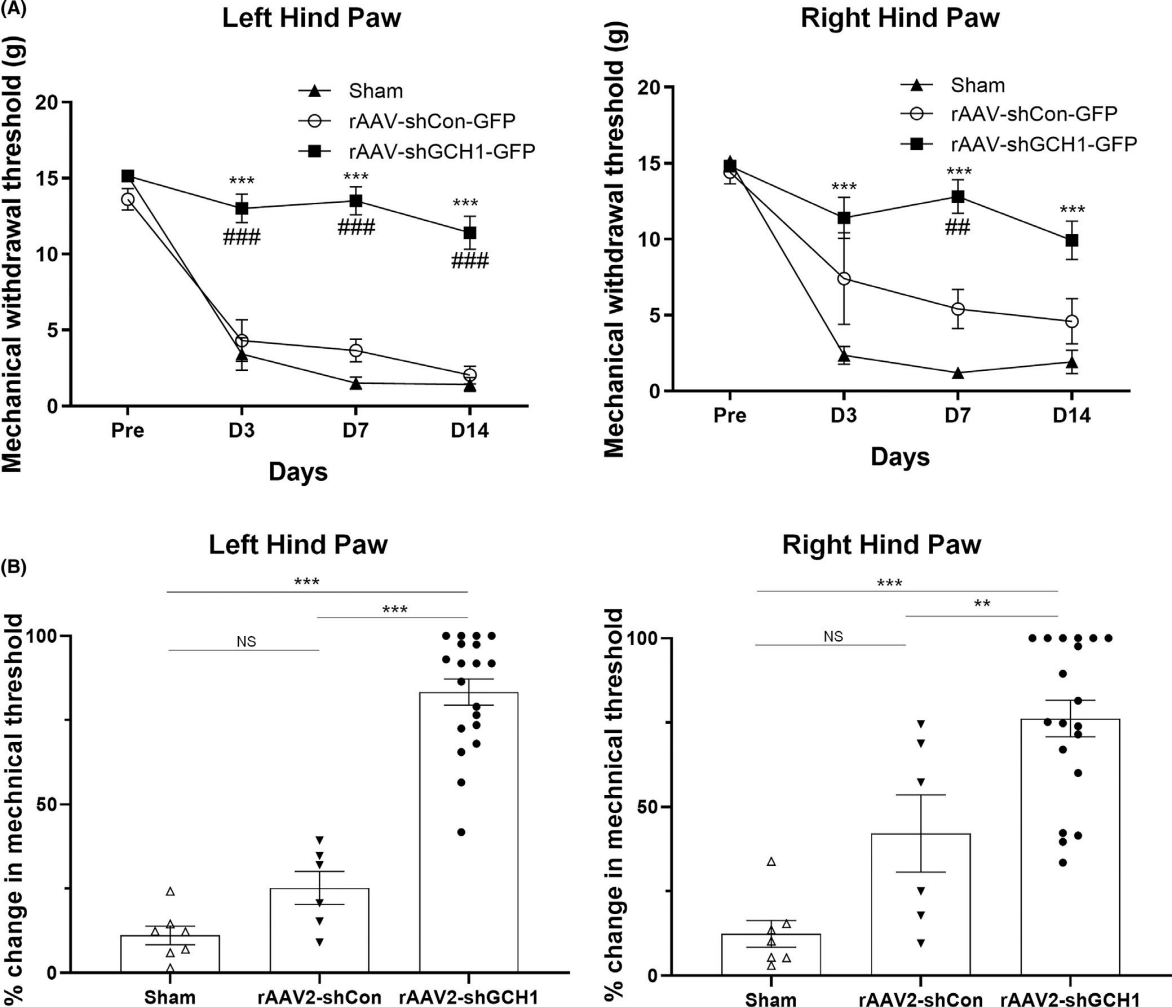
Behavioral testing after rAAV‐shGHC1 injection in a STT lesion model. A, Mechanical withdrawal threshold of rAAV‐shGCH1‐GFP (*n* = 19), rAAV‐shCon‐GFP (*n* = 6) and sham group (*n* = 7) was monitored using Von Frey filaments on postoperative day 3, 7, and 14 for both left and right hind paw. The mechanical withdrawal threshold of rAAV‐shGCH1‐injected group significantly differed from the sham group (*** *p* < 0.001) and rAAV‐shCon‐GFP‐injected group (## *p *< 0.01, ### *p* < 0.001) Two‐way ANOVA followed by Bonferroni post‐hoc analysis was performed to evaluate differences between groups. B, Changes in paw withdrawal thresholds after injecting rAAV‐shGCH1‐GFP, sham and rAAV‐shCon‐GFP. Mechanical thresholds in the rAAV‐shGCH1‐GFP‐injected group were significantly different from those in the sham group and control group (rAAV‐shCon‐GFP) in the left hind paw (rAAV‐shGCH1‐GFP: 83.34 ± 3.8%, rAAV‐shCon‐GFP: 25.17 ± 4.8% and Sham group: 11.12 ± 2.7%) and right hind paw (rAAV‐shGCH1‐GFP: 76.22 ± 5.3%, rAAVshCon‐GFP: 42.15 ± 11.4 and Sham group: 12.41 ± 3.9). **p < 0.01, ***p < 0.001

It is well known that mechanical allodynia occurs bilaterally in the hind paws as early as 3 days after a unilateral STT lesion.^16^, ^21^ The mean mechanical threshold for the normal left hind paw was 13.6 g, and it decreased to 4.31 g, 3.66 g, and 2.05 g on postoperative days 3, 7, and 14, respectively, after STT lesioning and rAAV‐shCON‐GFP injection. Sham group also showed decreased to 3.49 g, 1.52 g, and 1.43 g throughout the experiment. However, when we injected the rats with rAAV‐shGCH1‐GFP after STT lesioning, the hind paw mean mechanical thresholds on postoperative days 3, 7, and 14 were 13 g, 13.5 g, and 11.4 g, respectively (with a 15.1‐g baseline mean threshold). These differences in left hind paw between rAAV‐shCON‐GFP and rAAV‐shGCH1‐GFP injections were statistically significant (day 3, *p* < 0.001; day 7, *p* < 0.001; day 14, *p* < 0.001) (Figure 3A).

The contralateral side (right hind paw) was also evaluated after STT lesioning and injection. The mean mechanical threshold for the normal right hind paw was 14.4 g, and it decreased to 7.4 g, 5.4 g, and 4.59 g on postoperative days 3, 7, and 14, respectively, after STT lesioning and rAAV‐shCON‐GFP injection. Sham group had a similar level of mechanical threshold as shCON group (2.36 g, 1.2 g, and 2.5g). Following rAAV‐shGCH1‐GFP injection after STT lesioning, the mean mechanical thresholds on postoperative days 3, 7, and 14 were 11.4 g, 12.8 g, and 9.92 g, respectively (with a 14.8‐g baseline mean threshold). A trend was noted for the right hind paw threshold differences between rAAV‐shCON‐GFP and rAAV‐shGCH1‐GFP injections; however, only postoperative day 7 data showed a statistically significant change (*p* < 0.01, Figure 3B).

### 3.3 Immunofluorescence of Iba1 and GFAP in the STT lesion model with rAAV‐shGCH1‐GFP or rAAV‐shCON‐GFP injection in the dorsal horn

**FIGURE 4:**
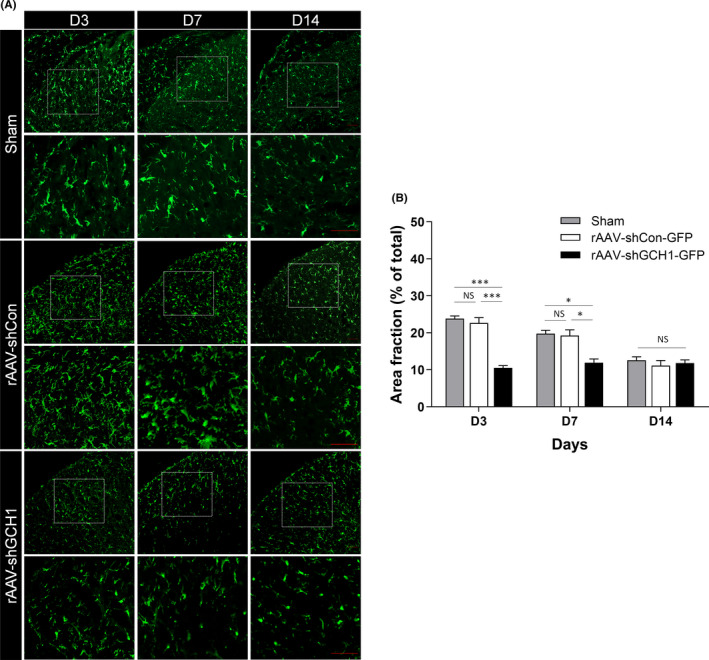
Neuroprotective effect of shGCH1 in the STT lesion model. A, Immunofluorescence of Iba1 in the spinal cord (coronal sections) on postoperative days 3, 7, and 14 after rAAV injections. Scale bars = 50 μm. Microglial activation is evident on days 3 and 7 after SCI with rAAV‐shCon‐GFP injection or Sham group, whereas activation decreased in the ipsilateral dorsal horn lasting until day 14 after rAAV‐shGCH1‐GFP injection. B, The difference in microglial activation between the three groups was statistically significant. (on day 3, ###*p* < 0.001 versus Sham group and ***p < 0.001 versus rAAV‐shCon‐GFP; at day 7, #*p* < 0.05 versus Sham group and *p < 0.05 versus rAAV‐shCon‐GFP)

After SCI, microglial activation is noted in the dorsal horn, and its activation is a well‐known key process for NP development.^22^ Microglial activation was reduced on days 3 and 7 after rAAV‐shGCH1‐GFP injection in the ipsilateral dorsal horn compared with that after rAAV‐shCON‐GFP injection, and this difference in activation between the three groups was statistically significant both on postoperative days 3 (*p* < 0.001) and 7 (*p* < 0.01) (Figure 4).

**FIGURE 5:**
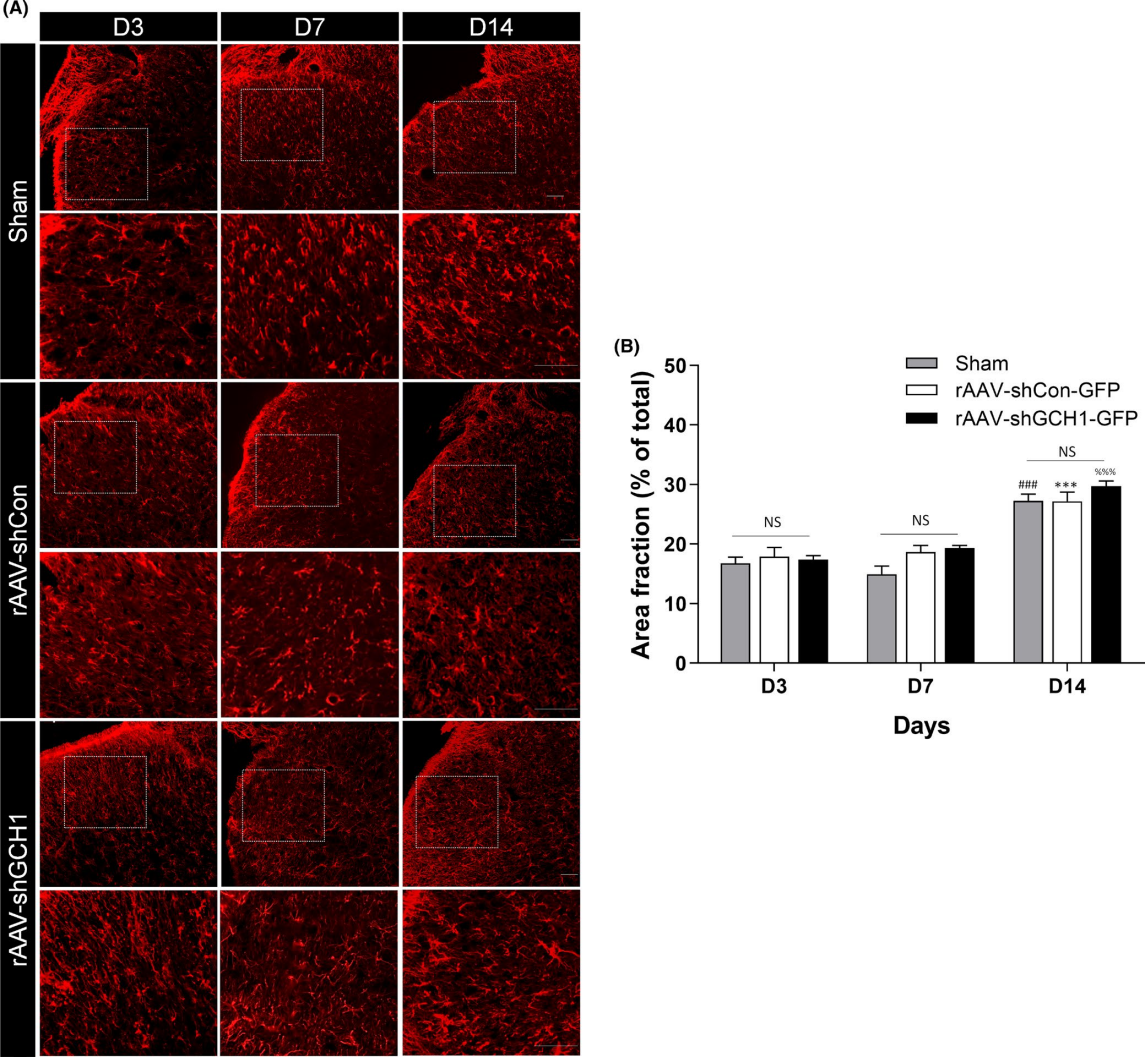
The activation of astrocyte in the STT lesion model. A, Immunofluorescence of GFAP in the spinal cord (coronal sections) on postoperative days 3, 7, and 14 after rAAV injections. Scale bars = 50 μm. Astrocytic activation is evident in all three groups at day 14 after SCI regardless of the treatment. The relative expression of astrocyte (B) The astrocyte activation showed a similar pattern between groups

In line with the role of microglia and astrocyte in STT studied by Naseri et al.^23^ immunofluorescence of GFAP in spinal cord on postoperative day 3, 7, and 14 were expressed more significantly on day 14 in all three groups (Sham, ### *p* < 0.001; rAAV‐shCON,*** *p* < 0.001; rAAV‐shGCH1, %%% *p* < 0.001), and there were no other changes by injecting shGCH1 (Figure 5).

## 4 DISCUSSION

Chronic central NP following SCI is a common complication, and once developed, it is often severe and affects the daily quality of life. Several pharmacological, neurosurgical, and behavioral treatment options are available; however, only one‐third of patients experience satisfactory pain relief. It is thought that the lack of understanding of the mechanisms underlying CNP after SCI leads to these insufficient management results.

Central sensitization after neuronal hyperexcitability in the dorsal horn, supraspinal changes, and gliosis within the spinal cord and elsewhere are suggested mechanisms for the development of NP after SCI.^24^, ^25^, ^26^, ^27^, ^28^, ^29^, ^30^ There have been several recent reports of microglial activation contributing to the development and maintenance of NP after nervous system injury; these reports have used various peripheral nerve injury models (eg, partial sciatic nerve ligation, sciatic nerve inflammation, spinal nerve ligation) and also a central model of contusive SCI, wherein after injection of a microglial inhibitor, NP‐related electrophysiological and behavioral responses were attenuated.^30^, ^31^, ^32^, ^33^, ^34^ Our previous efforts to downregulate GCH1 by injecting rAAV‐shGCH1 into the sciatic nerve in a peripheral NP model showed attenuated microglial activation.^12^ It is well known that after peripheral nerve injury, primary afferent fibers are abnormally activated, leading to microglial activation and recruitment, and these activated microglia participate in central sensitization by increasing the activity of second‐order nociceptive neurons in the dorsal horn.^23^, ^35^, ^36^, ^37^ We, therefore, attempted to downregulate microglial activation by directly injecting rAAV2‐shGCH1‐GFP into the dorsal horn in an early stage of SCI. STT lesioning model has unique characteristics, such as early onset of pain response to mechanical stimuli without affecting motor weakness below the lesioned spine level, which does not affect the measurement of mechanical withdrawal threshold.^16^ After STT lesioning, significant expression change of Iba1 till 7 days after surgery and of GFAP after day 14 are well described.^23^ In our rAAV‐shGCH1‐GFP injection group, microglia was significantly deactivated comparing with sham STT group or rAAV‐shCON‐GFP injected group till 14 days, and GFAP staining did not showed any significant changes among groups during test periods. Alleviation of NP behavior was prompt and lasted for 2 weeks after injection. This timing is important because this injection effect is correlated with the known immediate activation of microglia and lasts only for 2 weeks. Other later developing mechanisms of NP are suspected after that time.

BH4 is an essential cofactor for norepinephrine, dopamine, serotonin, and nitric oxide, all of which have prominent roles in pain signaling.^8^, ^9^, ^10^ In sensory neurons, excessive BH4 increases pain sensitivity. By directly regulating the rate‐limiting enzyme GCH1, BH4 levels may be reduced and may lead to pain modulation. Woof et al.^38^ showed the cellular localization of GCH1 by BH4 production in a peripheral nerve injury model. Except in the dorsal horn, each pathway related to BH4 production was decreased in the sciatic nerve and in the dorsal root ganglion. Because BH4 also exists in the brain, liver, and endothelial cells, systemic SPR inhibition was selected, and sepiapterin accumulation was suggested as a sensitive biomarker. Although excessive BH4 is important for pain development, we focused on the deactivation of microglia in the dorsal horn as a strategy for alleviating central NP using rAAV‐GCH1 injection. The behavioral test was performed for 4 weeks after injections in both groups (data not shown), and the early pain modulation effect by deactivating microglia was not sustained, suggesting further complexity in the development of NP. After SCI, GABAergic inhibitory interneurons are decreased not only in number but also in their activity in the superficial dorsal horn (laminae I‐III), which could account for the decreased activity of GAD 65 and 67.^39^ Thus, another suggestion for the long‐term attenuation of NP could be the restoration of GABAergic tone or the prevention of GABAergic neuronal loss by combining the modulation of GAD genes.

This study had some limitations. First, this was only a short‐term study of how rAAV‐shGCH1 injection could affect microglial deactivation and the progression of NP after SCI. Also, because of declination of microglial response to an insult with aging and of sexual dimorphisms under pathological conditions, it is still unclear that this injection could be beneficial to all populations.^40^, ^41^, ^42^, ^43^ Second, BH4 levels were not examined after rAAV‐shGCH1 injection. This approach represents a first attempt to modulate the difficult clinical problem of NP after SCI by injecting rAAV‐shGCH1 in the dorsal horn. In most clinical SCI cases after trauma, a spinal fusion operation is needed immediately. This represents a potential window of opportunity for the application of therapeutics such as rAAV‐GCH1. Hence, further studies using contusive SCI models should be performed to assess the possibility of microglial deactivation by rAAV‐GCH1 injection, and not only morphological analysis of microglial deactivation but also quantifying several pro‐inflammatory cytokines and chemokines released by microglia are needed to reveal the neuromodulatory effect of rAAV‐GCH1 injection in dorsal horn of SCI models.^44^

## 5 CONCLUSION

We demonstrated that NP after SCI could be modulated bilaterally by deactivating microglial activation after a unilateral dorsal horn injection of rAAV‐shGCH1 in a STT lesion SCI model. This new attempt would be another therapeutic approach for NP after SCI, which once happens; there is no clear curative options still now.

## CONFLICT OF INTEREST

The authors declare that they have no competing interests.

## Supporting information

Supplementary Material

## Data Availability

The data that support the findings of this study are available from the corresponding author upon reasonable request.
