# Supplementary figures and images for: Microglial deactivation by adeno‐associated virus expressing small‐hairpin GCH1 has protective effects against neuropathic pain development in a spinothalamic tract‐lesion model
Source: CNS Neurosci Ther. 2021 Nov 29;28(1):36–45. doi: 10.1111/cns.13751 (PMC8673712; doi:10.1111/cns.13751)

Full unedited bot for Fig. 2

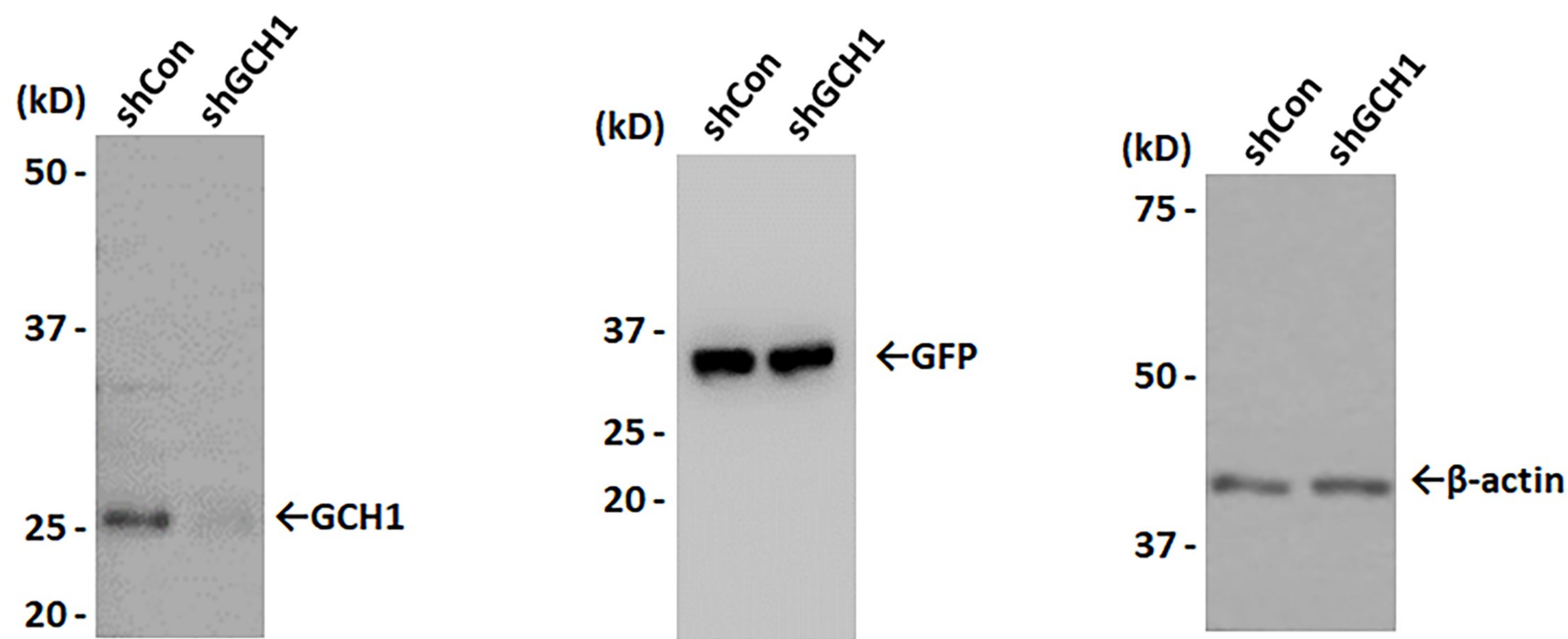

Supplement: Supplementary file 1 — Supplementary Material [file CNS-28-36-s001.pdf]
